# A Comparative Study on the Predictive Value of Different Resting-State Functional Magnetic Resonance Imaging Parameters in Preclinical Alzheimer's Disease

**DOI:** 10.3389/fpsyt.2021.626332

**Published:** 2021-06-11

**Authors:** Sheng-Min Wang, Nak-Young Kim, Dong Woo Kang, Yoo Hyun Um, Hae-Ran Na, Young Sup Woo, Chang Uk Lee, Won-Myong Bahk, Hyun Kook Lim

**Affiliations:** ^1^Department of Psychiatry, College of Medicine, Catholic University of Korea, Seoul, South Korea; ^2^Department of Psychiatry, Keyo Hospital, Uiwang, South Korea

**Keywords:** function, magnetic resonance imaging, diagnosis, Alzheimer's disease, amyloid

## Abstract

**Objective:** Diverse resting-state functional magnetic resonance imaging (rs-fMRI) studies showed that rs-fMRI might be able to reflect the earliest detrimental effect of cerebral beta-amyloid (Aβ) pathology. However, no previous studies specifically compared the predictive value of different rs-fMRI parameters in preclinical AD.

**Methods:** A total of 106 cognitively normal adults (Aβ+ group = 66 and Aβ− group = 40) were included. Three different rs-fMRI parameter maps including functional connectivity (FC), fractional amplitude of low-frequency fluctuations (fALFF), and regional homogeneity (ReHo) were calculated. Receiver operating characteristic (ROC) curve analyses were utilized to compare classification performance of the three rs-fMRI parameters.

**Results:** FC maps showed the best classifying performance in ROC curve analysis (AUC, 0.915, *p* < 0.001). Good but weaker performance was achieved by using ReHo maps (AUC, 0.836, *p* < 0.001) and fALFF maps (AUC, 0.804, *p* < 0.001). The brain regions showing the greatest discriminative power included the left angular gyrus for FC, left anterior cingulate for ReHo, and left middle frontal gyrus for fALFF. However, among the three measurements, ROI-based FC was the only measure showing group difference in voxel-wise analysis.

**Conclusion:** Our results strengthen the idea that rs-fMRI might be sensitive to earlier changes in spontaneous brain activity and FC in response to cerebral Aβ retention. However, further longitudinal studies with larger sample sizes are needed to confirm their utility in predicting the risk of AD.

## Introduction

Alzheimer's disease (AD) is a progressive brain disorder characterized by cognitive impairment, behavioral disturbance, and loss of daily functioning (1). Beta-amyloid (Aβ) plaques and neurofibrillary tangles of misfolded tau protein are known to play important roles in the development and progression of AD (2). The pathophysiological Aβ process may begin many years before the onset of dementia (3). Thus, increasing research focus on this long preclinical phase of AD may provide a critical opportunity for early therapeutic intervention and secondary prevention (4, 5).

Aβ pathology can be assessed using a cerebrospinal fluid (CSF) Aβ42 assay or amyloid positron emission tomography (PET) imaging (6). However, a CSF study is relatively invasive (7), and a PET scan, besides being cost-intensive, is still not available in some countries (8). In the biomarker model of AD, cerebral Aβ accumulation is necessary but not sufficient to produce clinical symptoms of mild cognitive impairment (MCI) and dementia (9, 10). Studies suggested that synaptic dysfunction and neurodegeneration could be the earliest product of cerebral Aβ accumulation and may be an important pathophysiological pathway leading to symptom presentation (11, 12). Moreover, recent evidence further showed that early synaptic dysfunction assessed by functional magnetic resonance imaging (fMRI) may be detected even before tau-mediated neuronal injury observed in FDG-PET and volumetric loss found in structural MRI (13).

Studies using resting-state fMRI (rs-fMRI) have facilitated our understanding of AD pathophysiology based on its intrinsic activity (14). Mounting evidence showed that a network of brain regions that together constitute the default mode network (DMN) highly overlap with the spatial distribution of early amyloid pathology (15). In addition, research investigating the effect of amyloid burden on rs-fMRI have repeatedly demonstrated decreased functional connectivity (FC) of the DMN from the posterior portion [precuneus, posterior cingulate cortex (PCC)] to the anterior portion [anterior cingulate cortex (ACC)] and from the precuneus to hippocampus (15–17).

Analytic approaches of rs-fMRI can be broadly divided into functional integration and functional segregation methods (18, 19). The functional integration method focuses on the functional relationship by analyzing rs-fMRI connectivity, while the functional segregation method focuses on the local function of specific brain regions by analyzing rs-fMRI activity (20). Seed-based correlational analysis, which is one of the functional integration approaches, was the first method applied to rs-fMRI (21). It is also called region-of-interest (ROI)-based FC analysis because it is based on the activity in an *a priori*-defined ROI (the seed region), either a volume or a single voxel, which is compared to that of other voxels in the brain (22). In terms of functional segregation approaches, amplitude of low frequency fluctuations (ALFF) or fractional ALFF (fALFF) and regional homogeneity (ReHo) are methods commonly used. Both ALFF and fALFF methods measure total power of blood oxygen level–dependent (BOLD) signal within the low-frequency range between 0.01 and 0.1 Hz. In the fALFF, power within the low-frequency range (0.01–0.1 Hz) is divided by the total power in the entire detectable frequency range, so it is regarded as less sensitive to physiological noise than ALFF (23). Moreover, it is known to reflect the intensity of spontaneous neural activity. In contrast, ReHo has been suggested to demonstrate localized connectivity by measuring the synchrony of adjacent brain regions (20). By computing the Kendall coefficient of concordance (KCC) of the BOLD time-series, it represents a voxel-based measure of the similarity between the time-series of a single voxel and its nearest neighbors (24).

Multiple studies already revealed altered FC, fALFF, and ReHo maps in various brain regions, mainly, of the DMN in preclinical AD (14, 25). However, previous studies mainly focused on localizing alterations based on group-level differences between cognitively normal older adults with Aβ+ and Aβ−, and whether the group differences can be applied as diagnostic markers distinguishing Aβ+ subjects from Aβ− subjects is still unclear. We previously showed that aberrance of regional functional synchronizations within the DMN quantified using ReHo have significant sensitivity and specificity for discriminating between the Aβ+ and Aβ− groups (26). However, to the best of our knowledge, no previous studies specifically compared the predictive value of different rs-fMRI parameters in patients with preclinical AD. Thus, we aimed to further our previous research and investigate which rs-fMRI parameter among ROI-based FC, fALFF, and ReHo achieves the best discrimination between cognitively older adults with Aβ+ and Aβ−.

## Materials and Methods

### Subjects

A total of 106 elderly subjects with normal cognitive function were included in the study. All subjects were recruited from normal control volunteers of the Catholic Aging Brain Imaging (CABI) database, which contains brain scans of outpatients at the Catholic Brain Health Center, Yeouido St Mary's Hospital, The Catholic University of Korea from 2017 to 2019. The inclusion criteria were as follows: (1) subjects aged 60 years or more; (2) Mini-Mental Status Examination score of ≥27; (3) global Clinical Dementia Rating (CDR) score of 0 (27). The exclusion criteria were as follows: patients (1) having presumptive diagnosis of dementia, mild cognitive impairment (MCI), or other neurological or medical conditions which cause cognitive dysfunction (e.g., hypothyroidism); (2) with a history or current diagnosis of other psychiatric disorders (e.g., schizophrenia, delusional disorder, and substance abuse); (3) having unstable medical conditions (e.g., poorly controlled hypertension, angina, or diabetes); and (4) taking any psychotropic medications (e.g., antidepressants, benzodiazepines, and antipsychotics).

Subjects completed a self-report health questionnaire containing demographic data and medical history. The questionnaire was reviewed to confirm whether patients met the inclusion or exclusion criteria. In addition, a cognitive function assessment using the Korean version of the Consortium to Establish a Registry for Alzheimer's Disease (CERAD-K) was conducted within 4 weeks from the day they received an MRI scan. The CERAD-K included Verbal Fluency (VF), 15-item Boston Naming Test (BNT), Mini-Mental Status Examination (MMSE), Word List Memory (WLM), Word List Recall (WLR), Word List Recognition (WLRc), Constructional Praxis (CP), and Constructional Recall (CR) tests (28). This study was conducted in accordance with the ethical and safety guidelines set forth by the Institutional Review Board of the Catholic University of Korea, and all subjects provided written informed consent.

### PET Acquisition

^18^F-Flutemetamol (FMM) was produced, and FMM-PET data were collected and analyzed as described previously (29). The MRI of each participant was used to co-register, define the ROIs, and correct partial volume effects arising from expanding cerebrospinal spaces accompanying cerebral atrophy. We used a standardized uptake value ratio (SUVR) 90 min post-injection to analyze the FMM-PET data using the pons ROI as the reference. Global Aβ burden was expressed as the average of SUVR of the mean for the six cortical ROIs including the frontal, superior parietal, lateral temporal, striatum, ACC, and PCC/precuneus regions. A PET scan was conducted within 4 weeks of the clinical screening and cognitive function test. We used a cut-off for “high” or “low” neocortical SUVR of 0.62, consistent with cut-off values used in previous FMM PET studies (29).

### MRI Acquisition

MRI data were acquired by the Department of Radiology, Yeouido St. Mary's Hospital, The Catholic University of Korea, with a 3T Siemens MAGETOM Skyra machine and an eight-channel Siemens head coil (Siemens Medical Solutions, Erlangen, Germany). We utilized the following parameters for the T1-weighted volumetric magnetization-prepared rapid gradient echo scan sequences: TE = 2.6 ms, TR = 1,940 ms, inversion time = 979 ms, FOV = 230 mm, matrix = 256 × 256, and voxel size =1.0 × 1.0 × 1.0 mm^3^. In terms of rs-fMRI, they were collected using a T2^*^-weighted gradient echo sequence with TR = 2,000 ms, TE = 30 ms, matrix = 128 × 128 × 29, and voxel size = 1 × 1 × 2 mm^3^. One hundred and fifty volumes were acquired over 5 min while participants were instructed to “keep your eyes closed and think of nothing in particular.”

### Data Analysis

#### fMRI Data Processing

Rs-fMRI data preprocessing was carried out using Data Processing Assistant for Resting-State fMRI (DPARSF) (30), which is based on Statistical Parametric Mapping 12 (SPM12, http://www.fil.ion.ucl.ac.uk/spm). Slice timing and realignment for motion corrections were performed on the images. We excluded subjects with excessive head motion (cumulative translation or rotation > 2 mm or 2°), and framewise displacement (FD) was compared between the groups to prevent group-related differences from micro-head motion. The two groups did not show significant differences in mean FD scores (*P* > 0.05, two-sample *t*-tests), and the mean FD scores were used as covariates in group comparisons. In terms of spatial normalization, we utilized the International Consortium for Brain Mapping (ICBM) template (resampling voxel size = 3 mm × 3 mm × 3 mm) which was fitted to the “East-Asian brain.”

We further processed our functional data to make them fit for FC, fALFF, and ReHo analysis through DPARSF (30). In terms of FC, seed-based correlation analysis was conducted to explore the FC of the DMN. We used a spherical ROI (radius = 10 mm) centered at the given Montreal Neurological Institute (MNI) coordinates [0, −52, 30] located in the PCC/precuneus area as the seed for the FC analysis. The individual preprocessed data were bandpass-filtered at 0.01–0.1 Hz. The fMRI time series data were extracted from each PCC/precuneus seed in the filtered data, and then Pearson's correlation coefficients were calculated between the PCC/precuneus time series and the time series of all other voxels in the brain. We used Fisher's r-to-z transformation to transform the correlation coefficient at each voxel to a *z*-value. The resultant PCC/precuneus FC map for each participant was entered into the group level analysis.

To measure regional intrinsic brain activities in the resting state, fALFF and ReHo were computed using the individual preprocessed data. fALFF is the ratio between the sum of Fourier amplitudes within a specific low-frequency range (0.01–0.1 Hz) and the sum of Fourier amplitudes across the entire frequency range (0–0.2 Hz) (23). Fast Fourier Transform (FFT) was used to transform time series of each voxel to the frequency domain and to obtain a power spectrum. Then the power spectrum obtained by FFT was square-rooted and then averaged across 0.01–0.08 Hz at each voxel, which is defined as ALFF. The fraction of ALFF in a given frequency band to the ALFF over the entire frequency range yielded fALFF. This fALFF calculation was repeated for each voxel in the whole brain to create a fALFF map for each participant, which was entered into the group level analysis.

In terms of ReHo analysis, we used a similar procedure as described in detail in our previous research (26). Briefly, we removed linear trends from the functional images. Thereafter, data were filtered with a temporal band-pass of 0.01–0.08 Hz, and ReHo maps of all participants were made via routine procedures of DPARSF. We set the basic cube to calculate KCC by 3 mm × 3 mm × 3 mm voxels, and temporal sequences of the neighboring 26 voxels were used to calculate the KCC of the central voxel, which was assigned as the ReHo value of the central voxel. An unsmoothed ReHo map was drawn by repeating this procedure for all the voxels. This raw ReHo map was smoothed by 6 mm of full width at half maximum (FWHM).

#### Voxel-Based Morphometry

SPM 12 was implanted with MATLAB R2019b for VBM processing. All anatomical images were first reoriented by coordinating the anterior commissure matching the *x, y, z* origin (0, 0, 0) with the orientation approximated to the MNI space. Thereafter, images were segmented into gray matter, white matter, and CSF partitions using the unified segmentation procedure by Ashburner and Friston (31). In terms of spatial normalization, we used the Diffeomorphic Anatomical Registration Through Exponentiated Lie Algebra (DARTEL) algorithm, which is known to have the advantage of maximizing the accuracy of localization by registering participants' structural images to an asymmetric T1-weighted template derived from the participants' structural images rather than from standard T1-weighted templates of different samples (32). Results were considered significant if they consisted of more than 15 neighboring voxels that surpassed an uncorrected threshold of *p* < 0.005.

### Statistical Analysis

We used Statistical Package for Social Sciences software (SPSS, version 19, Chicago, IL) for the statistical analysis of baseline demographic and clinical variables. The differences between the Aβ+ and the Aβ− groups for continuous and categorical variables were analyzed using independent *t*-test and χ^2^ test, respectively. All statistical analyses used a two-tailed level of 0.05 for defining statistical significance. The general linear model (GLM) was used for measuring within and between group differences of the FC, fALFF, and ReHo maps. To examine relationships between Aβ deposition and ReHo in the Aβ+ group, the global mean SUVR value from the five ROIs were correlated with the voxel-wise ReHo maps of the brain using GLM. Statistical inferences were made at *p* < 0.05 (corrected for multiple comparisons using the false discovery rate at the voxel level) or *p* < 0.005 (uncorrected for the voxel level). Lastly, classification performance was assessed by computing sensitivity, specificity, positive predictive value, negative predictive value, and accuracy. The receiver operating characteristic (ROC) curve was utilized to calculate the area under the ROC curve (AUC). We used the Youden index to obtain the optimal cut-point value in the ROC analysis.

## Results

### Demographic and Clinical Characteristics

A total of 106 cognitively normal patients (Aβ+ group = 66 and Aβ− group = 40) were included in the study. Demographic and clinical data including age, education, gender, CDR score, and CERAD-K score did not significantly differ between Aβ− and Aβ+ groups. The Aβ+ group showed significantly higher Aβ retention on average in the ACC, frontal lobe, parietal lobe, precuneus, PCC, and temporal lobe compared with the Aβ− group (Table 1).

**Table 1:** Demographic and clinical characteristics of the study participants.

	**Aβ– group**	**Aβ+ group**	***P-*value**
	**(*N* = 66)**	**(*N* = 40)**	
Age (years ± SD)	71.62 ± 8.69	70.53 ± 7.47	0.493
Education (years ± SD)	12.35 ± 4.76	11.40 ± 5.35	0.350
Gender (M:F)	22:44	14:26	0.861
CDR (SD)	0	0	
**Regional FMM SUVR**			
Average	0.53 ± 0.028	0.73 ±.098	<0.01^*^
Anterior cingulate cortex	0.54 ± 0.042	0.73 ± 0.11	<0.01^*^
Frontal lobe	0.41 ±−0.036	0.65 ± 0.11	<0.01^*^
Parietal lobe	0.35 ± 0.044	0.55 ± 0.098	<0.01^*^
Precuneus	0.40 ± 0.047	0.68 ± 0.16	<0.01^*^
Posterior cingulate cortex	0.52 ±.038	0.79 ± 0.15	<0.01^*^
Temporal lobe	0.478 ± 0.09	0.66 ± 0.16	<0.01^*^
**CERAD-K battery (SD)**			
VF	16.25 ± 4.45	15.20 ± 4.30	0.242
BNT	12.54 ± 1.81	12.07 ± 2.46	0.308
MMSE	27.75 ± 2.11	27.45 ± 2.37	0.513
WLM	19.39 ± 3.57	18.65 ± 4.33	0.369
CP	10.59 ± 1.05	10.70 ± 0.94	0.586
WLR	6.21 ± 1.66	6.15 ± 1.98	0.868
WLRc	9.26 ± 0.95	7.70 ± 3.06	0.572
CR	7.70 ± 3.06	6.70 ± 2.96	0.100

* *false discovery rate corrected*.

*Aβ+, cognitively normal older adults with beta-amyloid retention; Aβ−, cognitively normal older adults without beta-amyloid retention; SD, standard deviation; BNT, Boston Naming Test; CDR, Clinical Dementia Rating; CERAD-K, the Korean version of the Consortium to Establish a Registry for Alzheimer's Disease; FMM, 18F-flutemetamol; CP, Constructional Praxis; CR, Constructional Recall; MMSE, Mini-Mental Status Examination; SUVR, standardized uptake value ratio; VF, Verbal Fluency; WLM, Word List Memory; WLR. Word List Recall; WLRc, Word List Recognition*.

### Group Comparison by Voxel-Wise Analysis

**Figure 1:**
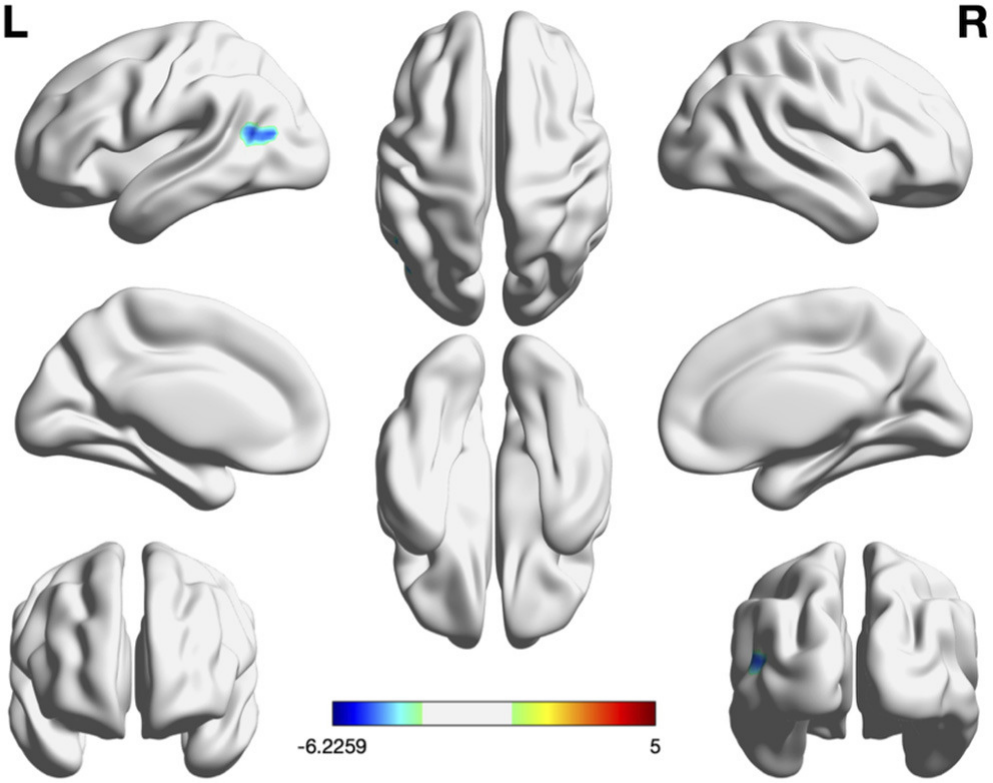
Significant regions in group comparison of FC, fALFF, and ReHo. In group comparison, cool color indicate regions showing lower values and warm color indicate regions showing higher values in the *A*β+ group than in *A*β–. The color bar indicates the *T*-score. Threshold: *p* < 0.05, false discovery rate-corrected at cluster level. Region-of-interest based FC was the only measure showing group difference in voxelwise analysis. Aβ+, cognitively normal older adults with beta amyloid retention; Aβ−, cognitively normal older adults without beta amyloid retention; fALFF, fractional amplitude of low-frequency fluctuations; FC, Functional connectivity; ReHo, regional homogeneity.

VBM analysis showed no significant group differences in the total intracranial volume, regional gray matter volume, and regional white matter volume. Compared with the Aβ− group, the Aβ+ group had significantly lower FC in the left angular gyrus (*p* < 0.05, FDR corrected; Table 2 and Figure 1). No group differences were noted for fALFF and ReHo, after correcting false discovery rate. However, the Aβ+ group showed lower fALFF values in the left precuneus, left middle frontal cortex, and right middle frontal cortex in uncorrected analysis (*p* < 0.005). In terms of ReHo, the Aβ+ group had higher values for the left superior temporal and right occipital-cuneus regions and lower values for the left ACC than the Aβ− group in uncorrected analysis (*p* < 0.005; Supplementary Table 1 and Supplementary Figure 1).

**Table 2:** Group comparison by voxel-wise analysis.

**Region**	**L/R**	**Cluster**	***T-*score**	***P-*value**	**MNI (*****x***,***y***,***z*****)**		
**FUNCTIONAL CONNECTIVITY**							
**Group differences**							
**Aβ+** **>** **Aβ−**							
None	N/A	N/A	N/A	N/A	N/A	N/A	
**Aβ+** **<** **Aβ−**							
Angular gyrus	L	52	5.23	<0.05^*^	−48	−63	15

* *False discovery rate-corrected at voxel level*.

*Aβ+, cognitively normal older adults with beta-amyloid retention; Aβ−, cognitively normal older adults without beta-amyloid retention*.

### Classifier Performance

**Figure 2:**
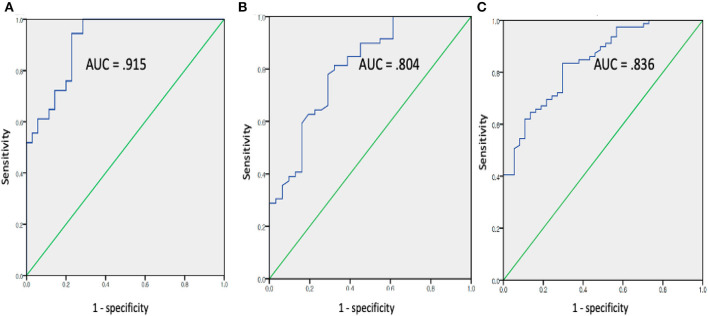
Receiver operating characteristic curve assessing results of mean FC **(A)**, fALFF **(B)**, and REHO **(C)** values in discriminating the *Aβ*+ from the *Aβ*- groups (*Aβ*+: cognitively normal order adults with beta amyloid retention, *Aβ*−: cognitively normal order adults with beta amyloid retention (fALFF, fractional amplitude of low-frequency fluctuations; ReHO, regional homogeneity).

The mean FC, fALFF, and ReHo values from the above ROIs showing group differences (FC: left angular gyrus; fALFF: left precuneus, left middle frontal cortex, and right middle frontal cortex; ReHo: left superior temporal, right occipital-cuneus regions, and left ACC) were used for ROC analysis in discriminating the Aβ+ group from the Aβ− group (Figure 2). The best discrimination was obtained when FC, between the left angular gyrus and PCC (the seed), was used with an AUC value of 0.915, sensitivity of 95.00%, specificity of 77.27%, positive predictive value of 72.70%, negative predictive value of 96.23, and accuracy of 83.96 (*P* < 0.001). For the fALFF measures, the left middle frontal gyrus resulted in good performance with an AUC value of 0.804, sensitivity of 77.5%, specificity of 71.1, positive predictive value of 62.0%, negative predictive value of 83.9, and accuracy of 73.58 (*P* < 0.001). For the ReHo values, the left ACC resulted in good performance with an AUC value of 0.836, sensitivity of 80.0%, specificity of 75.75%, positive predictive value of 66.67%, negative predictive value of 86.21, and accuracy of 77.36 (*P* < 0.001).

### Correlations Analysis Between FC and Aβ Deposition

We conducted correlation analysis between FMM retention and ROIs which showed group differences in voxel-wise analysis (FC values of the left angular gyrus) in the Aβ+ group alone. The results showed no correlation between FC values of the left angular gyrus with that of global mean FMM retention and six cortical regional FMM retentions including the frontal, superior parietal, lateral temporal, striatum, ACC, and PCC/precuneus areas.

## Discussion

To the best of our knowledge, this is the first study comparing the predictive value of different rs-fMRI features in differentiating cognitively normal older adults without Aβ retention from those with Aβ retention. Among the three measurements, ROI-based FC was the only measure showing group differences in voxel-wise analysis. No group differences were noted for fALFF and ReHo, after correcting the false discovery rate. FC showed the greatest accuracy in discriminating Aβ+ from Aβ− in cognitively normal older adults in the ROC curve analysis. Thus, our results suggested that FC might be a possible candidate biomarker distinguishing preclinical AD from the normal control.

It is generally acknowledged that ReHo and fALFF reflect local neural activity of the brain by manifesting the synchronization and amplitude of the BOLD signal, respectively (23, 33). In contrast, ROI-based FC analysis represents a spatial pattern of spontaneous activity on a global level (22). Thus, our findings support previous research which suggested that the brain is more appropriately studied as an integrated network rather than isolated clusters (20). Likewise, more consistent data are reported when cerebral Aβ pathology is studied by investigating the brain as an integrated network (i.e., ROI-based analysis using the PCC/precuneus as the seed) than by investigating isolated clusters (i.e., ReHo and fALFF analysis) (14, 25). We also found that regions showing significant group differences included the left angular gyrus, which is one of the most important functional hubs of the DMN (34). In line with our findings, multiple studies repeatedly showed that there is a large degree of convergence between decrement of DMN FC and cerebral Aβ deposition in cognitively normal older adults, and the convergence is most notable in areas including the angular gyrus, PCC/precuneus, and medial prefrontal cortex (16, 35).

Although no group differences were noted in the voxel-wise analysis, ROC analysis in discriminating the Aβ+ group from the Aβ− group was also conducted for fALFF and ReHo. FALFF also showed good discrimination performance. Particularly the Aβ+ group showed lower fALFF values in the bilateral middle frontal and left precuneus lesions than the Aβ− group. In line with our findings, rs-fMRI has consistently demonstrated decreased FC of the precuneus (17, 36). Furthermore, a more recent study showed that subjects with Aβ pathology had significantly lowered fALFF in the bilateral PCC and precuneus (37). Since the precuneus, along with the PCC, is well-known as an essential component of the DMN and brain network hub, it may be the earliest region showing the detrimental effect of Aβ cascade. On the other hand, our findings contradicted previous research by Zeng et al. (37), which showed that fALFF values in the inferior frontal gyrus (IFG) were higher in cognitively normal adults with Aβ+ than those with Aβ−. Zeng et al. speculated that, under the impact of Aβ pathology, increased neural activity in the IFG could be a compensational effect in an effort to maintain normal cognitive performance. Another study showed that higher activity and stronger FC in the IFG and insula may play an important role in protecting memory function against Aβ− associated pathology (38). Since Aβ+ patients of our study had lowered average MMSE scores than Aβ+ patients of Zeng et al.'s study (27.45 ± 2.37 vs. 28.60 ± 1.96), the compensatory increment of regional FC might not have been evident in our research. Further studies, with a larger sample size with longitudinal design, are needed to clarify this controversy.

The discriminating performance of ReHo was comparable to that of fALFF. However, the AUC in discriminating Aβ positivity was lower in the present research (AUC = 0.836) than in our previous study (AUC = 0.943) (26). This discrepancy could be attributed to the different ^18^F-labeled radiotracers used for Aβ imaging. Unlike our previous research, which used ^18^F-florbetaben (FBB), the present study used ^18^F-flutemetamol (FMM). In a recent head-to-head comparison study, FBB showed higher cortical uptake than FMM (39). Thus, FMM might have resulted in higher false negative values affecting the AUC. In addition, MRI acquisition parameters of the present study were different from that of our previous research. Thus, the scanner and parameter variability might have caused the discrepant results (40). In terms of regions showing significant group differences, similar to our previous findings (26), we found that the Aβ+ group had both increased and decreased ReHo values compared with the Aβ− group. Multiple studies highlighted that a mixed pattern of elevated and decreased activity is one of the obvious imaging features of AD pathology (41–43). Nonetheless, unlike most studies showing decreased activity mainly in the posterior region and increased activity in the anterior region, our results showed that the Aβ+ group had a significant ReHo decrease in the left anterior cingulate and increase in the left superior temporal and right occipital pole. Thus, our findings are in direct contradiction with the “age-related posterior-anterior theory,” which has been proven to be enhanced by the presence of AD pathology (44, 45). A longitudinal study by Cai et al. (46) showed that patients with mild cognitive impairment who reverted to normal, remained stable, or progressed to AD showed different patterns of ReHo values. Likewise, cognitively older adults with Aβ+ in our study might have been a heterogeneous group comprised of patients with diverse prognosis.

Both fALFF and ReHo represent regional neural activity, but no brain regions were either increased or decreased simultaneously in fALFF and ReHo. Mounting evidence suggested that the overlap in fALFF and ReHo represents that regions are not only active but are also active in synchronization with neighboring voxels (24). Thus, no brain regions were either activated or deactivated and engaged in a relatively large group of neurons at the same time. Future studies are needed to investigate whether such non-convergence between spectral and time-domain activities or fALFF and ReHo activities are important hallmarks of AD pathology.

Our results did not find significant correlations between FC of the left angular gyrus with that of regional or global amyloid deposition. Despite the consensus in the literature that amyloid pathology is related to a breakdown in functional brain networks, the association pattern between amyloid burden and FC is still controversial (47). In cognitively normal older adults, both positive and negative associations between FC and amyloid deposition were reported depending on the different anatomical regions (48, 49). However, additional studies containing a larger sample size are needed to determine whether FC patterns are associated with trajectories of amyloid pathology.

Our study contains multiple limitations. First, all data were collected from a single center limiting the generalizability of our results. Small sample size is another important issue. In addition, we reported *P* < 0.005 uncorrected for regions of fALFF and ReHo. Thus, these results must be interpreted cautiously because it may represent false positive results. Future studies with larger sample sizes are needed to confirm our findings. We were unable to describe clear neuropathological mechanisms explaining why three rs-fMRI analyses did not show overlapping brain regions. The cross-sectional design prevented us from making causal inferences. Not all patients with preclinical AD actually develop AD in the future, so our results cannot confirm that rs-fMRI can be a promising biomarker for AD. We were also unable to include the apolipoprotein epsilon4 (APOE4) allele, which is an important factor associated with neural activity and FC of the DMN in cognitively normal adults (50). Thus, longitudinal studies containing larger samples sizes with controlled genetic factors collected from multiple centers are needed to confirm our findings.

In conclusion, our results provide preliminary evidence that rs-fMRI might be helpful in distinguishing cognitively normal adults with cerebral Aβ retention from those without cerebral Aβ retention. Among three rs-fMRI parameters including ROI-based FC, fALFF, and ReHo, ROI-based FC provided the best discriminating performance. These results strengthen the idea that rs-fMRI might be sensitive to earlier changes in spontaneous brain activity and FC in response to Aβ retention. However, further longitudinal studies with a larger sample size are needed to confirm their utility in predicting the risk of AD.

## Data Availability Statement

The datasets generated for this study are available on request to the corresponding author.

## Ethics Statement

The studies involving human participants were reviewed and approved by Institutional Review Board of the Catholic University of Korea. The patients/participants provided their written informed consent to participate in this study. Written informed consent was obtained from the individual(s) for the publication of any potentially identifiable images or data included in this article.

## Author Contributions

S-MW and HL drafted the manuscript and contributed to project design, data collection, management, analysis, and interpretation. N-YK, YU, DK, and H-RN contributed to project design and data management. YW, CL, and W-MB contributed to study design and revision of manuscript. All authors contributed to the article and approved the submitted version.

## Conflict of Interest

The authors declare that the research was conducted in the absence of any commercial or financial relationships that could be construed as a potential conflict of interest.
