# Supplementary material for: A Comparative Study on the Predictive Value of Different Resting-State Functional Magnetic Resonance Imaging Parameters in Preclinical Alzheimer's Disease
Source: Front Psychiatry. 2021 Jun 11;12:626332. doi: 10.3389/fpsyt.2021.626332 (PMC8226028; doi:10.3389/fpsyt.2021.626332)
Supplement: Supplementary file 1 [file Table_1.DOCX]

| **Supplementary table 1. Group comparison by voxelwise analysis for fALFF and ReHo** | | | | | | | | | | |  |
| --- | --- | --- | --- | --- | --- | --- | --- | --- | --- | --- | --- |
| **fALFF** | | | | | | | | | | |  |
| Region | L/R | Cluster | T score | P value | | MNI (x,y.z) | | | | | |
| Group Differences |  |  |  | |  | |  | |  | |  |
| Aß+ > Aß- |  |  |  | |  | |  | |  | |  |
| None | N/A | N/A |  | N/A | | N/A | | N/A | | N/A | |
| Aß+ < Aß- |  |  |  | |  | |  | |  | |  |
| Precuneus | L | 42 | 4.10 | <0.005^+^ | | -12 | | -54 | | 51 | |
| Middle frontal | L | 94 | 4.62 | <0.005^+^ | | -33 | | 6 | | 45 | |
| Middle frontal | R | 36 | 3.70 | <0.005^+^ | | 30 | | 21 | | 39 | |
| **Whole brain Voxel wise ReHo analysis results** | | | | | | | | | | |  |
| Region | L/R | Cluster | T score | P value | | MNI (x,y.z) | | | | | |
| Group Differences |  |  |  | |  | |  | |  | |  |
| Aß+ > Aß- |  |  |  | |  | |  | |  | |  |
| Superior temporal | L | 101 | 3.17 | <0.005^+^ | | -69 | | -33 | | 9 | |
| Occipital pole and cuneus | R | 94 | 2.93 | <0.005^+^ | | 21 | | -99 | | 24 | |
| Aß+ < Aß- |  |  |  | |  | |  | |  | |  |
| Anterior Cingulate | L | 401 | 3.10 | <0.005^+^ | | -6 | | 45 | | 30 | |
| ^+^ uncorrected at voxel level  ReHo= regional homogeneity, Aß+= cognitively normal older adults with beta amyloid retention, Aß-= cognitively normal older adults without beta amyloid retention | | | | | | | | | | |  |
