# Supplementary material for: A Comparative Study on the Predictive Value of Different Resting-State Functional Magnetic Resonance Imaging Parameters in Preclinical Alzheimer's Disease
Source: Front Psychiatry. 2021 Jun 11;12:626332. doi: 10.3389/fpsyt.2021.626332 (PMC8226028; doi:10.3389/fpsyt.2021.626332)
Supplement: Supplementary file 2 [file Data_Sheet_1.PDF]

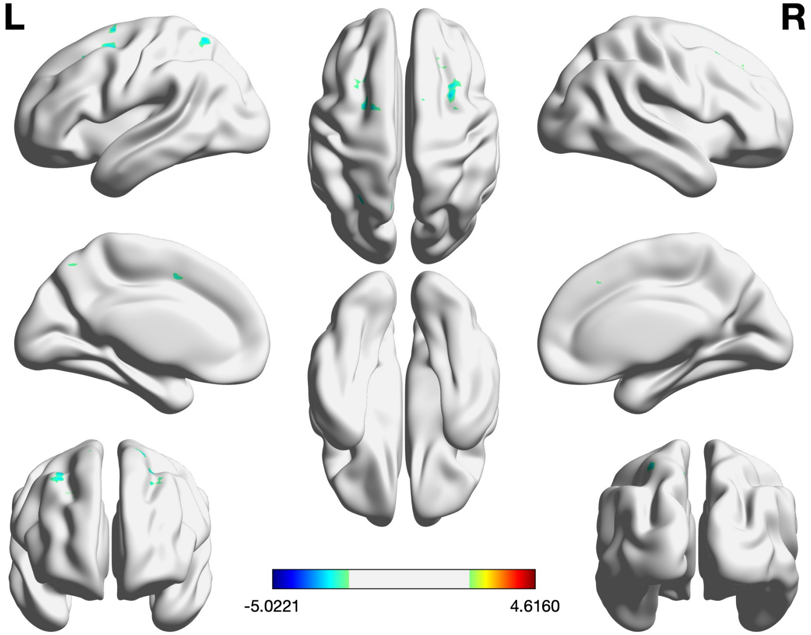


b. fALFF

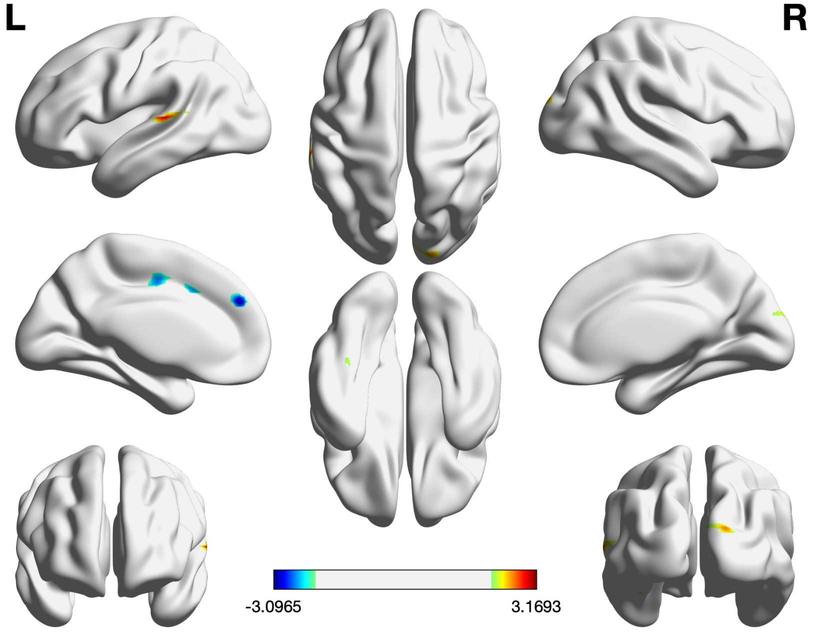


c. ReHo

Supplementary Fig 1. Significant regions in group comparison. a fALFF, b ReHo. In group comparison, cool color indicate regions showing lower values and warm color indicate regions showing higher values in the *Aß+* group than in *Aß-*. The color bar indicates the T-score. Threshold: $p < 0.005$, uncorrected at voxel level. *Aß+:* cognitively normal older adults with beta amyloid retention, Aß-: cognitively normal older adults without beta amyloid retention, fALFF: fractional amplitude of low-frequency fluctuations, ReHo: regional homogeneity
